# Medicine and surgery residents’ perspectives on the impact of COVID-19 on graduate medical education

**DOI:** 10.1080/10872981.2020.1818439

**Published:** 2020-09-13

**Authors:** Tanvi Rana, Christopher Hackett, Timothy Quezada, Abhishek Chaturvedi, Veli Bakalov, Jody Leonardo, Sandeep Rana

**Affiliations:** a Sidney Kimmel Medical College, Philadelphia, PA, USA; b Department of Neurology, Neuroscience Institute, Allegheny General Hospital, Allegheny Health Network, Pittsburgh, PA, USA; c Department of Medicine, Medicine Institute, Allegheny General Hospital, Allegheny Health Network, Pittsburgh, PA, USA; d Neurosurgery, Neuroscience Institute, Allegheny General Hospital, Allegheny Health Network, Pittsburgh, PA, USA

**Keywords:** COVID-19, pandemic, resident education, virtual learning, telemedicine, quarantine

## Abstract

The COVID-19 crisis has had an unprecedented impact on resident education and well-being: social distancing guidelines have limited patient volumes and forced virtual learning, while personal protective equipment (PPE) shortages, school/daycare closures, and visa restrictions have served as additional stressors. Our study aimed to analyze the effects of COVID-19 crisis-related stressors on residents’ professional and personal lives. In April 2020, we administered a survey to residents at a large academic hospital system in order to assess the impact of the pandemic on residency training after >6 weeks of a modified schedule. The primary outcome was to determine which factors or resident characteristics were related to stress during the pandemic. Our secondary goals were to examine which resident characteristics were related to survey responses. Data were analyzed with regression analyses. Ninety-six of 205 residents completed the survey (47% response rate). For our primary outcome, anxiety about PPE *(P *< 0.001), female gender (*P* = 0.03), and the interaction between female gender and anxiety about PPE *(P *= 0.04) were significantly related to increased stress during the COVID-19 pandemic. Secondary analyses suggested that medicine residents were more comfortable than surgical residents using telemedicine (*P* > 0.001). Additionally, compared to juniors, seniors believed that the pandemic was more disruptive, modified schedules were effective, and virtual *meetings* were less effective while virtual *lectures* were more effective (all *P* ≤ 0.05) Furthermore, the pandemic experience has allowed seniors in particular to feel more confident to lead in future health crises (*P* ≤ 0.05). Medicine and surgery residency programs should be cognizant of and closely monitor the effects of COVID-19 crisis-related factors on residents’ stress and anxiety levels. Transparent communication, telemedicine, online lectures/meetings, procedure simulations, advocacy groups, and wellness resources may help to mitigate some of the challenges posed by the pandemic.

## Introduction

Spring 2020 will be remembered as the beginning of the coronavirus disease-19 (COVID-19) outbreak in the United States. With a lack of viable treatment or immunization options for this virus, the public health response has centered around social distancing[1]. With ‘shelter-in-place’ orders and the cancellation of elective surgeries, the volume of patients coming to hospitals declined sharply [2,3]. Unsurprisingly, this has had a considerable impact on residency training programs across the country. Resident schedules have been modified to include virtual education, such as attending lectures through online platforms like Zoom, and clinic duties from home via telemedicine [4–6]. Nevertheless, residents continue to be on the front lines in the hospital where they treat patients with unknown COVID-19 statuses, which has understandably raised their anxiety levels [5,7]. Some residents have even been asked to self-quarantine if exposed to high-risk patients or if they themselves became ill [4,8].

To date, there is limited literature on how graduate medical education should be handled amid a pandemic and how residents evaluate the implementation of such contingency planning. Much of the guidance is based on the 2003 SARS epidemic [9,10], although the Accreditation Council for Graduate Medical Education (ACGME) has developed a framework for decision-making during the COVID-19 pandemic [11]. Additionally, a paper published by Chong et al. enumerated several guidelines for radiology programs to consider when responding to the impact of the COVID-19 crisis on their residents [12]; perspectives by Anderson et al [13], and DeFilippis et al [14]. noted educational challenges faced by graduate medical programs during this pandemic and potential solutions to address them; and a study conducted by Almarzooq et al. evaluated the experience that their cardiovascular fellowship program had using innovative virtual learning activities during this crisis [15].

Our survey study aims to better understand how the COVID-19 outbreak has impacted both the education and well-being of residents from various specialties at our institution. Specifically, we attempted to gain insight into resident perspectives regarding the effects of the crisis on their professional and personal lives, their views on the alternative learning opportunities implemented so far, and the extent to which they believe this unprecedented event will prepare them for the future.

## Methods

We aimed to better understand how the COVID-19 outbreak impacted educational experiences and well-being of residents at a large academic institution in Pittsburgh, Pennsylvania after >6 weeks of a modified schedule due to the pandemic. The Allegheny Health Network IRB determined this study to be exempt.

Authors TR (medical education research track at Sidney Kimmel Medical College), SR (neurology residency program director at Allegheny General Hospital [AGH]), and JL (neurosurgery residency program director at AGH) developed the survey with feedback from the neurology chief resident at AGH (TQ) and an internal medicine resident at AGH who has extensive research experience (VB). There were 25 to 31 questions, depending on if the resident: had been quarantined, was a U.S. citizen, had children, or was in a surgical residency. 15 questions were multiple choice, 9 were Likert-type scale, 6 were numerical scale (1–10), and 1 was open-ended. Given the limited literature on the subject matter, the survey did not have any prior testing. A minor participation incentive was awarded upon completion of the survey ($10 gift card).

Residents were contacted via email on 21 April 2020 to complete this anonymous, voluntary, online survey; the survey window closed on 3 May 2020. Statistical analyses were performed with SPSS (version 26.0, IBM Corp.). Descriptive statistics were assessed and reported as mean±standard deviation for normally distributed variables, median (interquartile range [IQR]) for variables not normally distributed, and proportion (numerator ‘of’ denominator) for categorical variables. We conducted subgroup analyses with a one-way ANOVA to evaluate how disruptive the COVID-19 crisis was on the specialties surveyed in our study (psychiatry, internal and family medicine, neurology, neurosurgery, and general surgery). Additionally, Mann-Whitney tests were performed to evaluate differences in anxiety levels between junior international medical graduates (IMGs) and senior IMGs as well as to assess differences in anxiety levels related to childcare between male and female residents.

The primary outcome (stress during COVID-19 crisis) was evaluated using a linear stepwise multiple regression model. Assumptions were checked and multicollinearity was found between age and resident experience; thus, age was excluded from all regression analyses. Additionally, because descriptive statistics suggested an interaction between female gender and anxiety related to PPE, the interaction term was computed and then added to the model. Stepping criteria was set at α = 0.05 for entry and α = 0.10 for removal from the model. The following covariates were entered into the linear stepwise multiple regression model: gender (male or female); residency type (medical or surgical); experience (junior: PGY 1–2 or senior: PGY ≥3); race; quarantine status (quarantined at any point during crisis or never quarantined during crisis); citizenship (U.S. citizen or international student); anxiety related to personal protective equipment (PPE); and the interaction of female gender and anxiety related to PPE.

Preplanned secondary analyses were conducted with sequential logistic regressions in two blocks to evaluate survey questions based on the same resident characteristics inputted as covariates in the regression analysis above. We conducted five separate logistic regression analyses assessing how each of the resident characteristics were related to residents’ perception of telemedicine and virtual learning, the impact of COVID-19 on their residency training and personal lives, and their beliefs on how the pandemic would affect their future self and career. In each logistic regression, we entered all characteristics of the residents in the first block (gender, residency type, experience, race, quarantine status, citizenship), except when the characteristic of interest for the regression was the outcome variable. The second block progressed in stepwise fashion (stepping criteria was set at α = 0.05 for entry and α = 0.10 for removal from the model) using likelihood ratios. The sequential design allowed us to control for confounding effects of the other resident characteristics, meaning that variables entered in the second block would predict the resident characteristic of interest above and beyond any confounding variables entered in the first block.

*P* values ≤0.05 were considered statistically significant. Survey questions and scales are provided in the online appendix (**eTable1**).

## Results

Of the initial 205 residents invited to participate in the study, 96 of them completed the survey (47% response rate). Regarding demographics, participants were more equally represented in terms of gender, age, and experience; however, medicine residencies, U.S. citizens, and residents who had not been quarantined were fairly overrepresented. Complete respondent demographics are depicted in **Table 1**. Response rates among participating residency programs can be viewed in **Table 2**.

**Table 1.** Demographics of survey participants.

Characteristic	Total, n/96 (%)
**Gender**	
Male	48 (50)
Female	48 (50)
**Age**	
20 to <30	45 (47)
30 to <40	50 (52)
40 to <50	1 (1)
**Race**	
White	59 (61)
Black or African American	2 (2)
Asian	24 (25)
Other	11 (11)
**Citizenship**	
U.S. citizen	78 (81)
International (visa holder)	18 (19)
**Parent Status**	
Parent	22 (23)
Not a Parent	74 (77)
**Quarantine Status**	
Quarantined	16 (17)
Not Quarantined	80 (83)
**Residency Program**	
Internal Medicine	36 (38)
General Surgery	18 (19)
Neurology	15 (16)
Psychiatry	12 (13)
Neurosurgery	8 (8)
Family Medicine	7 (7)
**Experience Level**	
Junior (PGY-1 and −2)	51 (53)
Senior (PGY ≥ 3)	45 (47)

**Table 2.** Survey response rate by residency program.

Residency Program	Survey Respondents (*n* = 96)	Total Residents (*n* = 205)	Response Rate (%)
**Medicine Programs**			
Family Medicine	7	21	33
Internal Medicine	36	100	36
Psychiatry	12	14	86
Neurology	15	15	100
**Surgery Programs**			
General Surgery	18	41	44
Neurosurgery	8	14	57

Overall, 50% (48 of 96) of survey respondents believed that the COVID-19 crisis had a negative impact on their clinical experience, but that the experience could be made up in the future. Meanwhile, 20% (19 of 96) agreed that the outbreak had a negative impact on clinical experience, and the experience could not be made up in the future. Conversely, 8% (8 of 96) of study participants indicated that the COVID-19 crisis had a positive impact on their clinical experience, 18% (17 of 96) indicated that it had no impact on their clinical experience, and the remaining 4% (4 of 96) indicated an ‘other’ impact.

Among surgery residents specifically, 50% (13 of 26) believed that the COVID-19 crisis had a negative impact on their surgical training, but that the experience could be made up in the future; 35% (9 of 26) agreed that the outbreak had a negative impact on their surgical training, and the experience could not be made up in the future; and 15% (4 of 26) indicated ‘no impact’.

A one-way ANOVA suggested that there was a significant difference in disruption of training among the specialties surveyed during the COVID-19 crisis (*F*(4,91) = 4.53, *P* = 0.002). A Bonferroni correction for multiple comparisons suggested that there was an observable difference between the extent of training disruption reported by psychiatry residents (3.58 ± 2.31) compared to those reported by residents from internal and family medicine (5.98 ± 2.05) [*P* = 0.01], neurology (6.00 ± 2.00) [*P* = 0.05], general surgery (6.78 ± 2.34) [*P* = 0.001], and neurosurgery (6.75 ± 2.38) [*P* = 0.02].

Notably, on a scale of 1–10 (1 = no anxiety, 10 = extreme anxiety), junior IMGs experienced a higher anxiety level than senior IMGs (9 [8,10] vs. 6 [2,8]) [*P* = 0.003]. Using the same scale, female residents with children expressed higher levels of anxiety than their male counterparts (8 [7,9] vs. 5.5 [3,8.5]) in terms of getting childcare at home upon school and daycare closures, but the difference was not statistically significant [*P* = 0.33].

In addition, we found that 79% (76 of 96) of residents agreed that the modified schedules (example: one week ‘on,’ one week ‘off’) implemented by their residency programs to minimize exposure to direct patient contact were an effective way to handle resident education amid the outbreak. One survey respondent commented, ‘[o]ur department did a great job of quickly shifting to virtual learning and patient care such that it did not feel very stressful … our program director was beyond amazing at helping us adapt to the change.’ Some other suggestions from survey participants to further improve resident education amid the COVID-19 crisis included: ‘more structured study plans for downtime’; ‘more articles for us to read to help guide education goals’; and ‘suggest an alternative patient/HIPAA safe communication platform for clinical conferences.’

Furthermore, for study participants that experienced ‘work from home’ time as part of their modified schedules, the extra time outside of the hospital was spent reading academic journals (73%, 70 of 96), doing practice questions (70%, 67 of 96), completing other hospital work (68%, 65 of 96), working on research (46%, 44 of 96), and/or volunteering (4%, 4 of 96). One survey respondent noted, ‘ … you can argue this has been a blessing because I have used the time off to complete some research projects that have lingered for months.’

### Primary outcome

#### Stress during COVID-19 crisis

The overall linear multiple regression model was able to explain 37% of the variance in stress [*F*(3,92) = 19.59, *P* < 0.001]. Three variables significantly contributed to residents’ stress and were included in the final model: anxiety regarding PPE [*P* < 0.001]; gender, with females reporting more stress (5.98 ± 1.85) than males (4.63 ± 2.13) [*P* = 0.03]; and the interaction between gender and anxiety concerning PPE, as females expressed more anxiety (5.67 ± 2.60) than males (4.44 ± 2.25) regarding PPE [*P* = 0.04]. Complete regression results can be viewed in **Table 3**.

**Table 3.** Primary analysis of characteristics contributing to resident stress during COVID-19 crisis.

Variables	B (95% CI)	*P* value
**Included in the final regression model**		
Anxiety about PPE	0.46 (0.32–0.60)	<0.001
Gender (female)	0.77 (0.07–1.46)	0.03
Interaction (gender * anxiety about PPE)	−0.30 (−0.58 – −0.01)	0.04
**Not included in the final regression model**		
Type of residency (medicine or surgical)	–	0.15
Experience (junior or senior)	–	0.70
Race	–	0.37
Quarantine status	–	0.54
International student	–	0.55

### Secondary outcomes

#### Medicine vs. Surgery

A sequential logistic regression was performed to examine associations between survey responses and residency type (medicine or surgery). Overall, the final model was statistically significant [(*Χ*[2](8, *N* = 96) = 54.71, *P* < 0.001]. Medicine residents (2 [1,3)]) reported being more comfortable than surgical residents (4 [4,4]) using telemedicine after >6 weeks of adhering to a modified schedule, compared to before the COVID-19 crisis [*P* < 0.001]. No other significant associations were found to predict group membership. Complete regression results can be viewed in **eTable 2**.

#### Juniors vs. Seniors

A sequential logistic regression was performed to examine associations between survey responses and residency experience (juniors or seniors). Overall, the final model was statistically significant [(*Χ*[2](13, *N* = 96) = 42.78, *P* < 0.001]. Five survey questions were included in the logistic regression model that predicted residency experience. Seniors felt that the pandemic was more disruptive on residency training (6.13 ± 2.04) compared to juniors (5.69 ± 2.53) [*P* = 0.02], but more seniors (89%, 40 of 45) reported that modified schedules were effective compared to juniors (71%, 36 of 51) [*P* = 0.05]. Fewer seniors (4%, 2 of 45) reported virtual *meetings* to be more effective than in-person *meetings* compared to juniors (20%, 10 of 51) [*P* = 0.04]. Conversely, a greater proportion of seniors (82%, 37 of 45) reported virtual *lectures* to be more effective than in-person *lectures* when compared with juniors (55%, 28 of 51) [*P* = 0.002]. Finally, seniors (7.20 ± 1.79) reported feeling more confident to lead during a future clinical crisis compared to juniors (6.22 ± 1.84) [*P* = 0.003]. No other variables were found to be significantly different between junior and senior residents. Complete regression results can be viewed in **eTable 3**.

#### Male vs. Female

A sequential logistic regression was performed to examine associations between survey responses and gender (male or female). Overall, the final model was statistically significant [(*Χ*[2](7, *N* = 96) = 14.55, *P* = 0.04]. The analysis suggested that there were not any associations between survey questions entered into the model that could predict gender above and beyond other characteristics of the residents. Complete regression results can be viewed in **eTable 4**.

#### Quarantined vs. Not quarantined

A sequential logistic regression was performed to examine associations between survey responses and quarantine status (quarantined or not). Overall, the final model was not statistically significant [*Χ*[2](9, *N* = 96) = 15.27, *P* = 0.08]. Two survey questions were included in the final model. Quarantined residents reported that they were more comfortable with telemedicine now compared to before the COVID-19 crisis (1 [1,3]), in contrast to nonquarantined residents (3 [1,4]); however, this difference was not statistically significant [*P* = 0.06]. Additionally, a greater proportion of residents who were quarantined (94%, 15 of 16) wanted online lectures to continue compared to nonquarantined residents (63%, 50 of 80), although this too was not statistically significant [*P* = 0.07]. Complete regression results can be viewed in **eTable 5**.

## Discussion

The results of this survey study suggest that there are positive and negative outcomes to consider when assessing the impact of the COVID-19 crisis on graduate medical education. This experience has caused considerable stress and anxiety for many residents in numerous aspects of their lives, including the disruption of their training, the loss of clinical and operative experience, the fear of inadequate PPE, the newfound challenge of obtaining reliable childcare, and the uncertainty of visa statuses in times of crisis. However, as one survey respondent commented, ‘[w]e are doing the best we can in a bad situation.’

Positive outcomes of the crisis should not be overlooked: there has been an increased comfort level with telemedicine, online platforms have been used to conduct meetings and lectures, and extra time at home has provided an opportunity to read academic journals, complete board-style practice questions, and catch up on research projects. Moreover, many participants – especially seniors – reported that going through this pandemic will give them confidence to take on leadership roles in the event of a similar future crisis.

The cessation of elective surgery during this outbreak has decreased the number of opportunities for surgery residents to learn in the operating room [2,4], which likely explains why surgery residents in our study perceived their training to be disrupted to a greater degree than medicine residents. Although the result was not statistically significant (possibly due to confounding with other resident characteristics), we believe that it is still an important finding since there are feasible solutions to mitigate the loss of hands-on surgical education, including cadaver lab experiences and virtual applications like Touch Surgery [14,16–18].

Decreases in elective procedures have also led to fewer preoperative and postoperative appointments, and therefore fewer opportunities for surgery residents to engage with telemedicine [3]. This may explain why surgery residents – compared to medicine residents – were significantly less comfortable using telemedicine and why they were less inclined to continue virtual encounters in the future. Conversely, the cognitive nature of medicine specialties, particularly psychiatry, enables telemedicine to facilitate certain valuable patient encounters [19,20]; therefore, medicine residents may still garner meaningful clinical experience despite not being in the hospital. Furthermore, for quarantined residents, telemedicine played an especially important role: it allowed these residents to stay connected to patients and, hence, served as the main route of obtaining clinical experience while in isolation [19]. As one survey respondent pointed out, ‘ … [telemedicine] is still not as good as an in-person visit, but it is much better than not visiting at all.’

There are also interesting differences to consider between junior residents and senior residents. Seniors felt that the crisis was especially disruptive to their residency training, likely because they lost important opportunities to perform tasks more independently [21,22]; however, they were ultimately satisfied with their modified schedules during the crisis. Conversely, juniors reported less disruption on their training, which may be a reflection of the fact that they have more time as trainees to make up for the lost experience. However, juniors were overall less satisfied with their modified schedules.

Compared to seniors, juniors appeared to be less comfortable with online *lectures*; this may be explained by an increased need for educational guidance and accountability, which are best delivered through the nuances of in-person communication and body language [23]. On the other hand, senior residents found online *meetings* less effective than junior residents, which may again be explained by the more personal nature of in-person communication [23]. Compared to juniors, seniors are expected to assume more leadership responsibilities within their programs; as such, their presence and input is likely to be best appreciated by attending physicians through in-person *meetings* instead of virtual ones [21,22,24].

Among the IMGs in our study, there was a high level of anxiety related to visa statuses, which we believe may be due to recent federal policies limiting immigration and visa processing [25–27]. Interestingly, junior IMGs expressed more anxiety about their visa status compared to senior IMGs. Though this result was not statistically significant (likely due to small sample sizes), its implications are still worthy of consideration. Given the physician shortage in our country, senior IMGs close to graduating may have their anxiety tempered by the belief that their visas will continue to be renewed since they will soon be independently-practicing physicians – a valuable service desperately needed across the country, especially in times of crisis [25,28,29]. Further investigation of the unique challenges faced by IMGs is warranted. Nonetheless, residency programs should be mindful of additional stressors facing IMGs and ensure these residents have access to mental health, legal, and advocacy resources when necessary [8,30–32].

Many residents with children – both male and female – are now facing the heavy burden of finding childcare following the indefinite closure of daycares and schools across the country in response to the COVID-19 crisis [33,34]. As one study participant noted with regards to balancing childcare and remote learning, ‘[t]hings end up all having to be completed at night.’ Additional studies should be performed to further elucidate other unique pandemic-related stressors affecting residents with children. Importantly, the shortage of PPE throughout the country has also been a significant source of anxiety among residents, particularly for females, and numerous depressing headlines in recent weeks have almost certainly exacerbated the feelings [35–38]. Overall, residency programs should consider actively inquiring into these various challenges, maintaining a policy of transparent communication, and aiding residents in finding the most up-to-date resources to manage these issues [8,13,31–33,39,40]. These steps are particularly important to take since many participants believed that these additional stressors of the COVID-19 crisis are negatively influencing their ability to learn.

Our findings are limited by the implementation of this study at a single urban tertiary care center in Pittsburgh, Pennsylvania – a city only moderately affected by the COVID-19 crisis [41]. The small sample size of surgery residents and lack of representation from other specialties (e.g. anesthesia, OB/GYN, radiology, emergency medicine) limits the generalizability of the results, as does the ongoing nature of the pandemic since perspectives may evolve as the crisis wears on. Lastly, since data was collected via survey, the limitations associated with response bias must be considered. Future research might consider developing and evaluating contingency plans for graduate medical education within all specialties – both medical and surgical – in order to maximize education and minimize anxiety when the next crisis arrives.

## Conclusion

The COVID-19 pandemic has had an unprecedented impact on the education and well-being of residents. Residency programs should be mindful of and take actions to mitigate its negative consequences on residents’ professional and personal lives, including the loss of clinical and operative experience as well as the stress and anxiety related to PPE shortages, new childcare responsibilities, and visa status uncertainties. However, there are also positive outcomes to consider; importantly, many residents at our institution – particularly in medicine specialties – expressed more comfort with telemedicine and other online platforms, and they are willing to continue using them in the future. Additionally, this unique experience has bolstered the confidence of some residents to step up as leaders when the next crisis arrives.

## Supplementary Material

Supplemental Material
