# Supplementary material for: Medicine and surgery residents’ perspectives on the impact of COVID-19 on graduate medical education
Source: Med Educ Online. 2020 Sep 13;25(1):1818439. doi: 10.1080/10872981.2020.1818439 (PMC7534325; doi:10.1080/10872981.2020.1818439)
Supplement: Supplemental Material [file ZMEO_A_1818439_SM8690.docx]

**ONLINE APPENDIX**

**eTable 1:** Survey questionnaire

| **#** | **Survey Questions** |
| --- | --- |
| **1** | **Which is your residency program?**   - 1. Internal Medicine   2. Neurology   3. Neurosurgery   4. Family Medicine   5. Psychiatry   6. General Surgery |
| **2** | **What year are you in?**   - 1. PGY-1   2. PGY-2   3. PGY-3   4. PGY-4   5. PGY-5   6. PGY-6   7. PGY-7   8. Fellow |
| **3** | **What is your gender?**   - 1. Male   2. Female |
| **4** | **What is your age?**   - 1. 20 to <30   2. 30 to <40   3. 40 to <50   4. 50+ |
| **5** | **What is your race?**   - 1. White   2. Black or African American   3. American Indian or Alaska Native   4. Asian   5. Naive Hawaiian or Pacific Islander   6. Other |
| **6** | **Have you been quarantined at any point during the current COVID-19 crisis?**   - 1. Yes   2. No |
| **7** | **What was the reason for your quarantine?** *(displayed if “yes” selected for Q6)*   - 1. Tested positive for COVID-19   2. Contact with high-risk patient   3. Fever and/or upper respiratory symptoms   4. Travel to high-risk area   5. Other (text entry) |
| **8** | **On a scale of 1-10, how stressful/anxiety-provoking has the current COVID-19 crisis been for you?**   - 1. 1 = not at all stressful/anxiety-provoking   2. 10 = extremely stressful/anxiety-provoking |
| **9** | **If you are not a United States citizen or green card holder, on a scale of 1-10, to what extent has the current COVID-19 crisis generated anxiety about your visa status?** *(leave blank if N/A)*   - 1. 1 = no anxiety   2. 10 = extreme anxiety |
| **10** | **If you are a parent, on a scale of 1-10, to what extent have recent school/daycare closures in response to the current COVID-19 crisis generated anxiety about getting childcare?** *(leave blank if N/A)*   - 1. 1 = no anxiety   2. 10 = extreme anxiety |
| **11** | **On a scale of 1-10, to what extent did having appropriate and/or adequate PPE generate anxiety during your in-person patient encounters?**   - 1. 1 = no anxiety   2. 10 = extreme anxiety |
| **12** | **How do you think any stress/anxiety related to the current COVID-19 crisis has impacted your ability to learn?**   - 1. Extremely positive impact   2. Moderately positive impact   3. Slightly positive impact   4. Neutral   5. Slightly negative impact   6. Moderately negative impact   7. Extremely negative impact |
| **13** | **On a scale of 1-10, how disruptive do you think the current COVID-19 crisis has been on your residency training?**   - 1. 1 = not at all disruptive   2. 10 = extremely disruptive |
| **14** | **Do you think the modified schedules that your residency program implemented to minimize exposure to direct patient contact were an effective way to handle resident education amid the current COVID-19 crisis?**   1. Yes 2. No 3. Other (text entry) |
| **15** | **What impact has the current COVID-19 crisis had on your clinical experience?**   - 1. Positive impact   2. Negative impact, and the clinical experience cannot be made up in the future   3. Negative impact, but the clinical experience can be made up in the future   4. No impact   5. Other (text entry) |
| **16** | **How comfortable do you feel partaking in telemedicine NOW vs. prior to the current COVID-19 crisis?**   - 1. Significantly more comfortable now   2. Moderately more comfortable now   3. Slightly more comfortable now   4. Neutral   5. Slightly less comfortable now   6. Moderately less comfortable now   7. Significantly less comfortable now |
| **17** | **How would you evaluate the overall impact that telemedicine has had on your clinical experience?**   - 1. Overall extremely positive impact   2. Overall moderately positive impact   3. Overall slightly positive impact   4. Neutral   5. Overall slightly negative impact   6. Overall moderately negative impact   7. Overall extremely negative impact |
| **18** | **After the current COVID-19 crisis is over, would you want to continue performing certain patient encounters via telemedicine?**   - 1. Yes   2. No   3. Other (text entry) |
| **19** | **How would you compare your experience using online platforms (e.g. Zoom) for meetings vs. in-person meetings?**   - 1. Online meetings are more effective than in-person meetings   2. Online meetings are as effective as in-person meetings   3. Online meetings are less effective than in-person meetings |
| **20** | **After the current COVID-19 crisis is over, would you want to continue using online platforms (e.g. Zoom) for meetings?**   - 1. Yes   2. No   3. Other (text entry) |
| **21** | **How would you compare your experience using online platforms (e.g. Zoom) for lectures vs. attending lectures in person?**   - 1. Online lectures are more effective than attending lectures in person   2. Online lectures are as effective as attending lectures in person   3. Online lectures are less effective than attending lectures in person |
| **22** | **After the current COVID-19 crisis is over, would you want to continue using online platforms (e.g. Zoom) for lectures?**   - 1. Yes   2. No   3. Other (text entry) |
| **23** | **During your “work from home” time, did you do any of the following?** *(select all that apply)*   - 1. Read academic journals   2. Work on research   3. Complete other hospital work   4. Volunteer   5. Do practice questions |
| **24** | **If applicable, what impact has the current COVID-19 crisis had on your ability to conduct research?** *(leave blank if N/A)*   - 1. Extremely positive impact   2. Moderately positive impact   3. Slightly positive impact   4. Neutral   5. Slightly negative impact   6. Moderately negative impact   7. Extremely negative impact |
| **25** | **What impact has the current COVID-19 crisis had on your surgical training?** *(displayed if “neurosurgery” or “general surgery” is selected for Q1)*   - 1. Positive impact   2. Negative impact, and the surgical experience cannot be made up in the future   3. Negative impact, but the surgical experience can be made up in the future   4. No impact   5. Other (text entry) |
| **26** | **How has the cancellation of cadaver laboratory sessions affected your education?** *(displayed if “neurosurgery” is selected for Q1)*   - 1. Positive impact   2. Negative impact, and the experience cannot be made up in the future   3. Negative impact, but the experience can be made up in the future   4. No impact   5. Other (text entry) |
| **27** | **What effect do you think the rescheduling of board exams will have on your future performance on the exam?** *(displayed if “neurosurgery” is selected for Q1)*   - 1. Rescheduling will have a positive effect on my future performance   2. Rescheduling will have a negative effect on my future performance   3. No effect   4. Other (text entry) |
| **28** | **Do you think that going through the current COVID-19 crisis as a resident will have any positive impact on your future career?**   - 1. Yes   2. No   3. Maybe   4. Other (text entry) |
| **29** | **How effective do you think your ability to adapt to change is NOW vs. prior to the current COVID-19 crisis?**   - 1. I am able to adapt to change much more effectively now   2. I am able to adapt to change somewhat more effectively now   3. No change   4. I am able to adapt to change somewhat less effectively now   5. I am able to adapt to change much less effectively now |
| **30** | **On a scale of 1-10, how confident would you feel to take on a leadership role in the clinical setting if another similar crisis were to occur in the future?**   - 1. 1 = not at all confident   2. 10 = extremely confident |
| **31** | **Do you have any other comments or suggestions as to how your education is being managed during the COVID-19 crisis?**   - 1. Text entry |

**eTable 2:** Variables examined in logistic regression comparing medicine and surgical resident experiences during COVID-19 crisis. All values are mean±standard deviation, median (IQR), or n (%).

| **Variables** | **Medicine**  **(n = 70)** | **Surgical**  **(n = 26)** | **OR (95% CI)** | **P value** |
| --- | --- | --- | --- | --- |
| **Included in the final regression model** | | | | |
| Experience (senior) | 31 (44.3) | 14 (53.8) | 1.93 (0.47 – 7.89) | 0.36 |
| Gender (female) | 37 (52.3) | 11 (42.3) | 0.78 (0.19 – 3.15) | 0.73 |
| Race (Caucasian) | 36 (51.4) | 23 (88.5) | – | 0.11 |
| Quarantined (yes) | 13 (18.6) | 3 (11.5) | 0.42 (0.05 – 3.36) | 0.41 |
| International student (yes) | 16 (22.9) | 2 (7.8) | 0.65 (0.06 – 7.37) | 0.73 |
| Comfort with telemedicine now | 2 (1,3) | 4 (4,4) | 5.26 (2.50 – 11.07) | <0.001 |
| **Not included in the final regression model** | | | | |
| Stress impacted ability to learn | 5 (4,5) | 5 (4,6) | – | 0.97 |
| Disrupting residency training | 5.57±2.25 | 6.77±2.30 | – | 0.97 |
| Modified schedules effective (yes) | 63 (90) | 13 (50) | – | 0.08 |
| Impact on clinical experience | 3 (2,3) | 3 (3,3.25) | – | 0.84 |
| Telemedicine impact clinical experience | 3 (2,4) | 4 (4,4) | – | 0.44 |
| Continue telemedicine after crisis (yes) | 59 (84.3) | 13 (50) | – | 0.39 |
| Meeting type (virtual) | 10 (14.3) | 2 (7.7) | – | 0.66 |
| Continue virtual meetings (yes) | 53 (75.7) | 19 (73.1) | – | 0.57 |
| Lecture type (virtual) | 13 (18.6) | 3 (11.5) | – | 0.71 |
| Continue virtual lectures (yes) | 48 (68.6) | 17 (65.4) | – | 0.92 |
| Will have positive impact on career (yes) | 34 (48.6) | 7 (26.9) | – | 0.59 |
| Ability to adapt to change now | 2 (2,3) | 2 (2,3) | – | 0.91 |
| Confidence to lead in future crisis | 6.64±1.93 | 6.68±1.73 | – | 0.56 |

**eTable 3:** Variables examined in logistic regression comparing junior and senior resident experiences during COVID-19 crisis. All values are mean±standard deviation, median (IQR), or n (%).

| **Variables** | **Junior**  **(n = 51)** | **Senior**  **(n = 45)** | **OR (95% CI)** | **P value** |
| --- | --- | --- | --- | --- |
| **Included in the final regression model** | | | | |
| Type (surgical) | 12 (23.5) | 14 (31.1) | 2.09 (0.56 – 15.09) | 0.21 |
| Gender (female) | 37 (52.3) | 11 (42.3) | 0.45 (0.15 – 1.38) | 0.16 |
| Race (Caucasian) | 29 (56.9) | 29 (66.7) | – | 0.27 |
| Quarantined (yes) | 8 (15.7) | 8 (17.8) | 1.03 (0.27 – 3.98) | 0.97 |
| International student (yes) | 7 (13.7) | 11 (24.4) | 3.41 (0.76 – 15.25) | 0.11 |
| Disrupting residency training | 5.69±2.53 | 6.13±2.04 | 1.37 (1.05 – 1.78) | 0.01 |
| Modified schedules effective (yes) | 36 (70.6) | 40 (88.9) | 8.56 (0.98 – 74.68) | 0.05 |
| Meeting type (virtual) | 10 (19.6) | 2 (4.4) | 0.19 (0.04 – 0.94) | 0.04 |
| Lecture type (virtual) | 7 (13.7) | 9 (20) | 14.89 (2.72 – 81.51) | 0.002 |
| Confidence to lead in future crisis | 6.22±1.84 | 7.20±1.79 | 1.48 (1.08 – 2.03) | 0.01 |
| **Not included in the final regression model** | | | | |
| Stress impacted ability to learn | 5 (4,6) | 5 (4,5) | – | 0.20 |
| Impact on clinical experience | 3 (3,3) | 3 (2,3) | – | 0.37 |
| Comfort with telemedicine now | 2 (1,4) | 2 (1,4) | – | 0.30 |
| Telemedicine impact clinical experience | 3 (2,4) | 3 (2 4) | – | 0.47 |
| Continue telemedicine after crisis (yes) | 36 (70.6) | 36 (80) | – | 0.19 |
| Continue virtual meetings (yes) | 36 (70.6) | 36 (80) | – | 0.89 |
| Continue virtual lectures (yes) | 32 (62.7) | 33 (73.3) | – | 0.09 |
| Will have positive impact on career (yes) | 23 (45.1) | 18 (40) | – | 0.46 |
| Ability to adapt to change now | 2 (2,3) | 2 (2,3) | – | 0.49 |

**eTable 4:** Variables examined in logistic regression comparing male and female resident experiences during COVID-19 crisis. All values are mean±standard deviation, median (IQR), or n (%).

| **Variables** | **Male**  **(n = 48)** | **Female**  **(n = 48)** | **OR (95% CI)** | **P value** |
| --- | --- | --- | --- | --- |
| **Included in the final regression model** | | |  |  |
| Type (surgical) | 15 (31.3) | 11 (22.9) | 0.52 (0.19 – 1.41) | 0.20 |
| Experience (senior) | 27 (56.3) | 18 (37.5) | 0.48 (0.20 – 1.17) | 0.11 |
| Race (Caucasian) | 27 (56.3) | 32 (66.7) | – | 0.75 |
| Quarantined (yes) | 8 (16.7) | 8 (16.7) | 0.81 (0.25 – 2.62) | 0.73 |
| International student (yes) | 14 (29.2) | 4 (8.3) | 0.27 (0.07 – 1.03) | 0.27 |
| **Not included in regression model** | | | | |
| Disrupting residency training | 6.08±2.31 | 5.71±2.32 | – | > 0.05 |
| Modified schedules effective (yes) | 37 (77.1) | 39 (81.3) | – | > 0.05 |
| Meeting type (virtual) | 2 (4.2) | 10 (20.8) | – | > 0.05 |
| Lecture type (virtual) | 6 (12.5) | 10 (20.8) | – | > 0.05 |
| Confidence to lead in future crisis | 6.88±1.98 | 6.48±1.76 | – | > 0.05 |
| Stress impacted ability to learn | 4.5 (4,5) | 5 (4,6) | – | > 0.05 |
| Impact on clinical experience | 3 (2,3) | 3 (2,3) | – | > 0.05 |
| Comfort with telemedicine now | 3 (1,4) | 2 (1,4) | – | > 0.05 |
| Telemedicine impact clinical experience | 4 (2,4) | 3 (2,4) | – | > 0.05 |
| Continue telemedicine after crisis (yes) | 34 (70.8) | 38 (79.2) | – | > 0.05 |
| Continue virtual meetings (yes) | 34 (70.8) | 38 (79.2) | – | > 0.05 |
| Continue virtual lectures (yes) | 32 (66.7) | 33 (68.8) | – | > 0.05 |
| Will have positive impact on career (yes) | 20 (41.7) | 21 (43.8) | – | > 0.05 |
| Ability to adapt to change now | 2 (2,3) | 2 (1,3) | – | > 0.05 |

**eTable 5:** Variables examined in logistic regression comparing quarantined resident versus non-quarantined resident experiences during COVID-19 crisis. All values are mean±standard deviation, median (IQR), or n (%).

| **Variables** | **Quarantined**  **(n = 16)** | **Not**  **Quarantined**  **(n = 80)** | **OR (95% CI)** | **P value** |
| --- | --- | --- | --- | --- |
| **Included in the final regression model** | | | | |
| Type (surgical) | 3 (18.8) | 23 (28.7) | 0.51 (0.07 – 3.92) | 0.52 |
| Experience (senior) | 8 (50) | 37 (46.3) | 1.29 (0.36 – 4.60) | 0.69 |
| Race (Caucasian) | 9 (56.3) | 50 (62.5) | – | 0.38 |
| Gender (female) | 8 (50) | 40 (50) | 0.91 (0.25 – 3.23) | 0.88 |
| International student (yes) | 4 (25) | 14 (17.5) | 0.85 (0.17 – 4.10) | 0.84 |
| Comfort with telemedicine now | 1 (1,3) | 3 (1,4) | 1.86 (0.98 – 3.52) | 0.06 |
| Continue virtual lectures (yes) | 15 (93.8) | 50 (62.5) | 7.09 (0.86 – 58.45) | 0.07 |
| **Not included in regression model** | | | | |
| Stress impacted ability to learn | 5 (4,6) | 5 (4, 5) | – | 0.69 |
| Disrupting residency training | 5.19±2.34 | 6.04±2.30 | – | 0.45 |
| Modified schedules effective (yes) | 13 (81.3) | 63 (78.8) | – | 0.13 |
| Impact on clinical experience | 3 (2,4) | 3 (2,3) | – | 0.54 |
| Telemedicine impact clinical experience | 2 (2, 4) | 3.5 (2,4) | – | 0.94 |
| Continue telemedicine after crisis (yes) | 15 (93.8) | 57 (71.3) | – | 0.30 |
| Meeting type (virtual) | 0 (0) | 12 (15) | – | 0.15 |
| Continue virtual meetings (yes) | 14 (87.5) | 58 (72.5) | – | 0.69 |
| Lecture type (virtual) | 2 (12.5) | 14 (17.5) | – | 0.65 |
| Will have positive impact on career (yes) | 7 (43.8) | 34 (42.5) | – | 0.73 |
| Ability to adapt to change now | 2 (2,3) | 2 (2,3) | – | 0.97 |
| Confidence to lead in future crisis | 6.94±2.14 | 6.63±1.82 | – | 0.99 |
